# Impact of CCL4 gene polymorphisms and environmental factors on oral cancer development and clinical characteristics

**DOI:** 10.18632/oncotarget.15615

**Published:** 2017-02-22

**Authors:** Ming-Yu Lien, Chiao-Wen Lin, Hsiao-Chi Tsai, Yng-Tay Chen, Ming-Hsui Tsai, Chun-Hung Hua, Shun-Fa Yang, Chih-Hsin Tang

**Affiliations:** ^1^ Graduate Institute of Basic Medical Science, China Medical University, Taichung, Taiwan; ^2^ Division of Hematology and Oncology, Department of Internal Medicine, China Medical University Hospital, Taichung, Taiwan; ^3^ Institute of Oral Sciences, Chung Shan Medical University, Taichung, Taiwan; ^4^ Department of Dentistry, Chung Shan Medical University Hospital, Taichung, Taiwan; ^5^ Department of Pediatrics, Medical Research and Medical Genetics, China Medical College Hospital, Taichung, Taiwan; ^6^ Department of Otolaryngology, China Medical University Hospital, Taichung, Taiwan; ^7^ Department of Otorhinolaryngology, China Medical University Hospital, Taichung, Taiwan; ^8^ Institute of Medicine, Chung Shan Medical University, Taichung, Taiwan; ^9^ Department of Medical Research, Chung Shan Medical University Hospital, Taichung, Taiwan; ^10^ Department of Biotechnology, College of Health Science, Asia University, Taichung, Taiwan

**Keywords:** oral cancer, CCL4, single-nucleotide polymorphism

## Abstract

In Taiwan, oral cancer has causally been associated with environmental carcinogens. CCL4 (C-C chemokine ligand 4), a macrophage inflammatory protein with a key role in inflammation and immune-regulation, was implicated in carcinogenesis by facilitating instability in the tumor environment. The purpose of this study was to identify gene polymorphisms of CCL4 specific to patients with oral squamous cell carcinoma (OSCC) susceptibility and clinicopathological characteristics. A total of 2,053 participants, including 1192 healthy people and 861 patients with oral cancer, were recruited for this study. Three single-nucleotide polymorphisms (SNPs) of the CCL4 gene were analyzed by a real-time PCR. We found that the T/T homozygotes of CCL4 rs1634507 G/T polymorphism and the GG haplotype of 2 CCL4 SNPs (rs1634507 and rs10491121) combined were associated with oral-cancer susceptibility. In addition, TA haplotype significantly decreased the risks for oral cancer by 0.118 fold. Among 1420 smokers, CCL4 polymorphisms carriers with the betel-nut chewing habit had a 15.476–20.247-fold greater risk of having oral cancer compared to CCL4 wild-type (WT) carriers without the betel-nut chewing habit. Finally, patients with oral cancer who had A/G heterozygotes of CCL4 rs10491121 A/G polymorphism showed a lower risk for an advanced tumor size (> T2) (*p*=0.046), compared to those patients with AA homozygotes. Our results suggest that the CCL4 rs1634507 SNP have potential predictive significance in oral carcinogenesis. Gene-environment interactions of CCL4 polymorphisms might influence oral-cancer susceptibility. CCL4 rs10491121 may be a factor to predict the tumor size in OSCC patients.

## INTRODUCTION

Oral cancer is one of the common malignant cancer in the head and neck region, more than 90% are squamous cell carcinomas (OSCCs) [1]. In Taiwan, it is the fourth most common male cancer and the fourth leading cause of male cancer death [2]. Despite combined with surgery, chemotherapy, and radiation, the prognosis and mortality of OSCC has remained poor [3]. The development of OSCC is mediated by accumulation of multiple genetic alterations and by environmental carcinogen-exposure influences. The alcohol, tobacco consumption, betel-quid chewing, chronic inflammation, and viral infection have been documented as risk factors for OSCC [4–6]. Single-nucleotide polymorphisms (SNPs), the most common type of DNA sequence variation influenced gene expression, protein function and disease susceptibility in particular individuals. SNP had potential predictive significance risk of oral cancer in previous studies [7–9]. Although the relationship between genetic polymorphisms and the environmental carcinogens was also reported [10, 11], identifying principal genes related to the susceptibility for OSCC is important for disease early detection.

CCL4 (C-C chemokine ligand 4) is well-known to be the macrophage inflammatory protein 1 (MIP-1β). CCL4 are belong to inflammatory CC chemokine subfamily located on chromosome 17 (q11-q21), which are secreted by specific cells after being triggered by antigens or mitogenic signals and attract additional cells involved in immune responses [12, 13]. CCL4 have also a second non-allelic copy, CCL4L (SCYA4L), that code for two highly similar proteins (>95% between CCL4 and CCL4L proteins). Roger *et al*. found SNP (rs4796195) in the intron 2 acceptor splice site displays a new complex splicing pattern [14]. This polymorphism generates two allelic variants: CCL4L1 (L1 allele) and new described variant, CCL4L2 (or L2 allele). L2 allele has been associated with HIV susceptibility in a case control study [14]. The recent study demonstrated that CCL4 serum concentration level was significantly higher in head and neck squamous cell carcinoma patients than in that of controls. CCL4 was also positive significantly associated with more-advanced disease stage [15].

These study findings suggest that CCL4 plays an important role in the oral cancer progression. High serum levels of CCL4 was documented associated with the presence of HCC in liver cirrhosis patients. CCL4 could bind to CCR5 (chemokine C-C motif receptor 5) in the carcinomatous tissues and Kupffer cells in the liver and induce the infiltration of inflammatory cells [16]. It was reported that highly expression of CCL4 was significantly positive correlation with lymph node metastasis in diffuse type gastric cancer and metastatic potential in prostate cancer [17, 18]. CCR5 was also found plays an important role in cancer progression through immune cell recruitment [19]. Over-expression of CCR5 in oral cancer patients might protect tissue cells from CCL5-induced carcinogenesis by decrease the levels of free CCL5, and CCR5 rs1799987 G/A SNP might associate with oral cancer risk [20].

Previous research observed that the T alleles and AT genotype of rs1719153 CCL4 SNP were more frequency in healthy controls than in HIV-1 positive patients [21, 22]. To our knowledge, none of studies determinate the impact of gene polymorphisms of CCL4 on the susceptibility of oral cancer. The current study analyzed relationships between SNPs rs1634507, rs10491121, and rs1719153 of the CCL4 gene and the risk of oral cancer. In addition, the influences of these SNPs combined with environmental carcinogens, including betel-nut and tobacco consumption, leading to a susceptibility to oral cancer, were evaluated. We also investigated the relationship between genetic variations and the clinicopathological characteristics of oral cancer. This is the first study to demonstrate a significant association between CCL4 polymorphisms and oral carcinogenesis.

## RESULTS

This study evaluated difference of demographic characteristics between 861 patients with oral cancer and 1192 normal controls (Table 1). Only male subjects are recruited to exclude the impact of gender differences on disease formation. The significant differences of age (*p* =0.031), betel quid chewing (*p* <0.001), cigarette smoking (*p* <0.001) and alcohol drinking (*p* <0.001) were observed. 427 (49.6%) patients had stage I and II and 434 (50.4%) patients had stage III+IV. Most tumors 737 (85.6%) were classified as moderately and poorly differentiated tumors

**Table 1:** Demographical characteristics in 1192 controls and 861 male patients with oral cancer

Variable	Controls n=1192(%)	Patients n=861(%)	*p* value
Age (yrs)	Mean ± S.D.	Mean ± S.D.	
	53.90 ± 10.01	54.92 ± 11.04	*p*=0.031
Betel quid chewing			
No	993 (83.3%)	170 (19.7%)	
Yes	199 (16.7%)	691 (80.3%)	*p* <0.001*
Cigarette smoking			
No	558 (46.8%)	86 (10.0%)	
Yes	634 (53.2%)	775 (90.0%)	*p* <0.001*
Alcohol drinking			
No	956 (80.2%)	390 (45.3%)	
Yes	236 (19.8%)	471 (54.7%)	*p* <0.001*
Stage			
I+II		427 (49.6%)	
III+IV		434 (50.4%)	
Tumor T status			
T1+T2		495 (57.5%)	
T3+T4		366 (42.5%)	
Lymph node status			
N0		582 (67.6%)	
N1+N2+N3		279 (32.4%)	
Metastasis			
M0		852 (99.0%)	
M1		9 (1.0%)	
Cell differentiation			
Well differentiated		124 (14.4%)	
Moderately or poorly differentiated		737 (85.6%)	

Mann-Whitney U test or Fisher's exact test was used between healthy controls and patients with oral cancer. * p value < 0.05 as statistically significant.

To estimate the potential influence of CCL4 gene polymorphisms on the development of oral cancer, three non-synonymous single-nucleotide polymorphisms (nsSNPs), rs1634507, rs10491121, and rs1719153 were evaluated in this investigation. In the controls, the genotypic frequency of CCL4 SNP rs1634507, rs10491121, and rs1719153 conformed the Hardy-Weinberg equilibrium (*p* = 0.09, χ^2^ value: 2.83; *p* = 0.82, χ^2^ value: 0.05; and *p* = 0.21, χ^2^ value: 1.57, respectively).Moreover, in the case group, the frequencies of three selected SNPs also met the Hardy-Weinberg equilibrium (*p* = 0.71, χ^2^ value: 0.14; *p* = 0.87, χ^2^ value: 0.03; and *p* = 0.63, χ^2^ value: 0.23, respectively).

Genotype distributions and associations between oral cancer and CCL4 gene polymorphisms are shown in Table 2. Alleles with the highest distribution frequency for the rs1634507, rs10491121, and rs1719153 genes of CCL4 in both of our recruited male oral-cancer patients and healthy controls were respectively homozygous for G/G, heterozygous for A/G, and homozygous for A/A. People with T/T homozygotes of CCL4 rs1634507 G/T polymorphism had a 1.479-fold (95% CI: 1.073–2.040; *P* = 0.017) significantly higher risk of developing oral cancer compared to those with G/G homozygotes after adjusting confound factors. However, there were no significant differences in the incidences of oral cancer in individuals with the rs10491121, and rs1719153 genes polymorphisms of the CCL4 gene compared to wild-type (WT) individuals.

**Table 2:** Odds ratio (OR) and 95% confidence interval (CI) of oral cancer associated with CCL4 genotypic frequencies

Variable	Controls n=1192(%)	Patients n=861 (%)	OR (95% CI)	*p* value
**rs1634507**				
GG	585 (49.1%)	391 (45.4%)	1.000 (reference)	
GT	518 (43.5%)	382 (44.4%)	1.103 (0.918-1.326)	*p*=0.295
TT	89 (7.4%)	88 (10.2%)	1.479 (1.073-2.040)	*p*=0.017*
GT+TT	607 (50.9%)	470 (54.6%)	1.158 (0.972-1.381)	*p*=0.101
G allele	1688 (70.8%)	1164 (67.6%)	1.000 (reference)	
T allele	696 (29.2%)	558 (32.4%)	1.163 (1.017-1.329)	p=0.028*
**rs10491121**				
AA	307 (25.8%)	214 (24.9%)	1.000 (reference)	
AG	592 (49.7%)	428 (49.7%)	1.037 (0.837-1.285)	*p*=0.739
GG	293 (24.5%)	219 (25.4%)	1.072 (0.837-1.373)	*p*=0.580
AG+GG	885 (74.2%)	647 (75.1%)	1.049 (0.857-1.283)	*p*=0.644
A allele	1206 (50.6%)	856 (49.7%)	1.000 (reference)	
G allele	1178 (49.1%)	866 (50.3%)	1.036 (0.915-1.172)	*p=0.579*
**rs1719153**				
AA	541 (45.4%)	391 (45.4%)	1.000 (reference)	
AT	538 (45.1%)	383 (44.5%)	0.985 (0.819-1.185)	*p*=0.873
TT	113 (9.5%)	87 (10.1%)	1.066 (0.783-1.450)	*p*=0.686
AT+TT	651 (54.6%)	470 (54.6%)	0.999 (0.838-1.191)	*p*=0.991
A allele	1620 (68.0%)	1165 (67.7%)	1.000 (reference)	
T allele	764 (32.0%)	557 (32.3%)	1.014 (0.888-1.158)	*p=0.840*

The odds ratio (OR) with their 95% confidence intervals were estimated bylogistic regression models. * *p* value < 0.05 as statistically significant.

Interaction effects between environmental risk factors and genetic polymorphisms of CCL4 are shown in Table 3. Among 1420 smokers, subjects with at least one T allele of rs1634507, one G allele of rs10491121, one T allele of rs1719153, and the betel-nut-chewing habit had respective risks of 17.563-fold (95% CI: 11.856-26.018), 20.247-fold (95% CI: 12.075-33.949), and 15.476-fold (95% CI: 10.457-22.904) of developing oral cancer. Individuals with either at least one T allele of rs1634507, one G allele of rs10491121, one T allele of rs1719153 or who chewed betel nut had respective risks of 4.976-fold (95% CI: 3.508-7.057), 3.576-fold (95% CI: 2.162-5.913), and 5.123-fold (95% CI: 3.591-7.308) of developing oral cancer compared to individuals with WT homozygotes who did not chew betel nut. According to the above results, we suggest that CCL4 gene polymorphisms have a strong impact on oral-cancer susceptibility in smoking consumers and/or betel-nut.

**Table 3:** Associations of the combined effect of CCL4 gene polymorphisms and betel nut chewing with the susceptibility to oral cancer among 1420 smokers

Variable	Controls n=634 (%)	Patients n=775 (%)	OR (95% CI)	AOR (95% CI)
**rs1634507**				
GG genotype & non-betel nut chewing	208 (32.8%)	47 (06.1%)	1.000	1.000 (reference)
GT or TT genotype or betel nut chewing	336 (53.0%)	375 (48.4%)	**4.939 (3.484-7.002)**	**4.976 (3.508-7.057)**
GT or TT genotype with betel nut chewing	90 (14.2%)	353 (45.5%)	**17.357 (11.727-25.690)**	**17.563 (11.856-26.018)**
**rs10491121**				
AA genotype & non-betel nut chewing	106 (16.7%)	20 (02.6%)	1.000	1.000 (reference)
AG or GG genotype or betel nut chewing	396 (62.5%)	265 (34.2%)	**3.546 (2.145-5.861)**	**3.576 (2.162-5.913)**
AG or GG genotype with betel nut chewing	132 (20.8%)	490 (63.2%)	**19.670 (11.753-32.921)**	**20.247 (12.075-33.949)**
**rs1719153**				
AA genotype & non-betel nut chewing	201 (31.7%)	45 (5.8%)	1.000	1.000 (reference)
AT or TT genotype or betel nut chewing	332 (52.4%)	381 (49.2%)	**5.126 (3.593-7.311)**	**5.123 (3.591-7.308)**
AT or TT genotype with betel nut chewing	101 (15.9%)	349 (45.0%)	**15.433 (10.430-22.836)**	**15.476 (10.457-22.904)**

The adjusted odds ratio (AOR) with their 95% confidence intervals was estimated by multiple logistic regression models after controlling for age.

To explore the effects of polymorphic genotypes of CCL4 on the clinical status of OSCC. AS shown in Table 4, for the genotypic frequencies of the SNPs, only CCL4 rs10491121 showed significant associations with clinical pathological variables in male oral cancer patients. Compared to the WT genotype, patients with A/G heterozygotes of CCL4 rs10491121 A/G polymorphism showed a significantly lower risk (*p*=0.046) for being at an advanced tumor size (> T2).

**Table 4:** Odds ratio (OR) and 95% confidence intervals (CI) of clinical status associated with genotypic frequencies of CCL4 rs10491121 in 861 male oral cancer patients

Variable			AOR (95% CI)	*p* value
Clinical Stage				
**rs10491121**	Stage I+IIn=427 (%)	Stage III+IVn=434 (%)		
AA	100 (23.4%)	114 (26.3%)	1.00	
AG	217 (50.8%)	211 (48.6%)	1.186 (0.853-1.649)	*p*=0.311
GG	110 (25.8%)	109 (25.1%)	1.161 (0.796-1.694)	*p*=0.439
Tumor size				
**rs10491121**	≦ T2n=495 (%)	> T2n=366 (%)		
AA	111 (22.4%)	103 (28.1%)	1.00	
AG	258 (52.1%)	170 (46.5%)	**0.714 (0.513-0.995)**	*p*=0.046*
GG	126 (25.5%)	93 (25.4%)	0.799 (0.547-1.168)	*p*=0.247
Lymph node metastasis				
**rs10491121**	Non=852 (%)	Yesn=9 (%)		
AA	212 (24.9%)	2 (22.2%)	1.00	
AG	423 (49.7%)	5 (55.6%)	1.254 (0.241-6.527)	*p*=0.788
GG	217 (25.4%)	2 (22.2%)	0.978 (0.136-7.010)	*p*=0.982
Cell differentiated grade				
**rs10491121**	≦Grade In=124 (%)	>Grade In=737 (%)		
AA	36 (29.0%)	178 (24.2%)	1.00	
AG	50 (40.3%)	378 (51.3%)	0.650 (0.490-1.035)	*p*=0.070
GG	38 (30.7%)	181(24.6%)	1.033 (0.626-1.705)	*p*=0.898

Cell differentiate grade: grade I: well differentiated; grade II: moderately differentiated; grade III: poorly differentiated.

The adjusted odds ratio (AOR) with their 95% confidence intervals were estimated by multiple logistic regression models after controlling for age.* *p* value < 0.05 as statistically significant.

**Figure 1:**
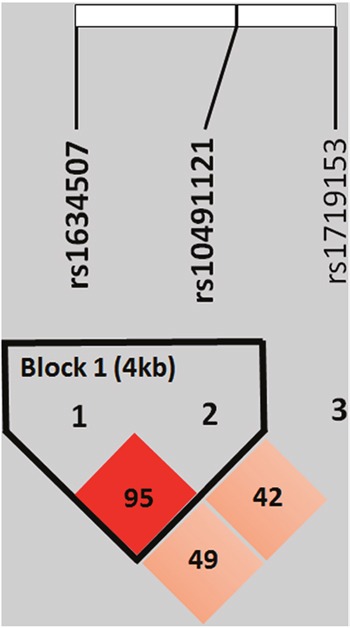
Linkage disequilibrium (LD) map for single nucleotide polymorphisms in the CCL4 gene There are 2053 participants, including 1192 healthy people and 861 patients with oral cancer, in this study. Block is pairwise *D*’ plots and haplotype blocks obtained from HAPLOVIEW.

We further explored the haplotypes to evaluate the combined effect of the three polymorphisms on oral-cancer susceptibility. The distribution frequencies of CCL4 rs1634507 and rs10491121 haplotypes in our recruited individuals were analyzed. The most common haplotype in the control was G/A (48.9%), and it was therefore chosen as a reference. Compared to the reference, CCL4 haplotypes, G/G, significantly increased the risks for oral cancer by 1.313 fold (95% CI: 1.110-1.553). Another CCL4 haplotypes, T/A significantly decreased the risks for oral cancer by 0.118 fold (95% CI: 0.035-0.400) (Table 5). The reconstructed linkage disequilibrium (LD) plot of the three SNPs is shown in Figure 1. We determined one observed haploblock in which rs1634507 and rs10491121 showed 95% linkage disequilibrium in our study.

**Table 5:** Odds ratio (OR) and 95% confidence interval (CI) of oral cancer associated with *CCL4* rs1634507/rs10491121 haplotype frequencies

Haplotype block	Controls n = 2384	Patients n = 1722	AOR (95% C.I.)
Block 1: rs1634507/rs10491121			
G/A	1165 (48.9%)	875 (50.8%)	1.000 (reference)
T/G	701 (29.4%)	531 (30.8%)	0.999 (0.866-1.153)
G/G	515 (21.6%)	297 (17.3%)	1.313 (1.110-1.553)*
T/A	3 (0.1%)	19 (1.1%)	0.118 (0.035-0.400)*

The adjusted odds ratio (AOR) with their 95% confidence intervals was estimated by multiple logistic regression models after controlling for age. * p value < 0.05 as statistically significant.

## DISCUSSION

The CCL4 played an important role in several cancers. Increased CCL4 expression in prostate cancer cell promoted tumor invasion and migration by modulating integrin pathway activation. *In vivo* studies showed that CCL4 increased tumor volume and angiogenesis [23]. Conversely highly level of CCL4 expression in esophageal squamous cell carcinoma (ESCC) was correlated with a more favorable prognosis, suggesting a role of CCL4 in recruitment of tumor infiltration CD8+ T lymphocytes and affect cancer microenvironment [24]. Thus, the biological function and significance of CCL4 expression in malignancy was still controversial. In this study, we first investigated whether polymorphisms within the CCL4 gene played a significant role in the development of oral cancer. We found that participants with T/T homozygotes of CCL4 rs1634507 G/T polymorphism (OR: 1.479; 95% CI: 1.073–2.040; P = 0.017) were significantly associated with increased oral cancer risk compared to those with G/G homozygotes after adjusting confound factors (Table 2). To our knowledge, this is first examination of CCL4 gene polymorphisms in oral cancer.

The CCL4 rs1634507 polymorphism in promoter regions was not related to HIV-1 infection but could be significantly associated with Acquired Immune Deficiency Syndrome (AIDS) more-rapid disease progression [22]. The gene variants could increase CCL4 levels, allowing the interaction with more CCR5 receptor molecules, promoting their internalization and thus helping entry restriction of HIV-1 R5 strains. Eva *et.al* found CCL4 secreted by tumor-infiltrating granulocytic and monocytic myeloid-derived suppressor cells (MO-MDSCs) recruit high numbers of regulatory T cells (Tregs) via CCR5 in lymphoma and melanoma [25]. CCL4 induced tumor growth through regulation of antitumor immune responses by a network of MDSCs and Tregs within the tumor microenvironment. The properties of tumor microenvironments have a strong impact on the disease progression, response to therapy and prognosis [26]. The gene polymorphisms may increase CCL4 levels and reduce antitumor immunity in tumor microenvironment. Consequently, enhance the risk of oral cancer with T allele of CCL4 *rs1634507* polymorphisms. Although the functional importance of the SNP had not been tested, a relationship with the risk of oral cancer is initiated based on the locations of the analyzed variants.

In the present study, higher rations of oral cancer patients used betel quid chewing, tobacco or alcohol were observed compared to the control group (Table 1). This finding showed that these environmental carcinogens are associated with increased risks for oral cancer. Long-term betel-quid chewing, tobacco, and alcohol were well identified strong risk factor for oral cancer [27, 28]. Tobacco and alcohol use might enhance DNA damage by mutation of *XRCC1* and *p53* genes [29]. Betel consumption was an important issue in Taiwan because of high oral habits prevalent in this population [5]. Arecoline, a major areca nut alkaloid might induce significantly increases expressions of β-catenin, Hypoxia inducible factor (HIF)-1α and Heat shock protein 70 (HSP70) in oral cancer [30–32].

Exposure to environmental carcinogen might result in earlier onset of oral cancer development. Furthermore, genomic changes progressively alter cellular phenotype and might more significantly lead cells to grow from the pre-neoplastic stage into malignancy [33]. The combination of polymorphism of nuclear factor kappa B1 (NFKB1) -94 ATTG, NFKBIA -826 T,-881 G alleles and environmental carcinogen were associated with increasing risk of betel-quid related oral cancer [34]. Polymorphisms of several genes were determined as being associated with the risk of oral cancer [20, 35]. It is distinct that genetic components may play a pivotal role in carcinogenesis. In our study, the synergistic effect of environmental factor (betel quid and smoking) and CCL4 gene SNPs (rs1634507, rs10491121, and rs1719153) on the risk of oral cancer (Tables 3) are well demonstrated. Among smoker people with GT or TT genotype of CCL4 rs1634507 polymorphism combined with betel nut chewing had a 17.563-fold (95% CI: 11.856-26.018) increased risk to develop oral cancer compared with those with G/G homozygotes. Also, AG or GG genotype of CCL4 rs10491121 polymorphism and AT or TT genotype of CCL4 rs1719153 polymorphism combined with betel nut chewing had a 20.247-fold (95% CI: 12.075-33.949) and a 15.476-fold (95% CI: 10.457-22.904) increased risk to progress oral cancer. The long-term exposure to cigarette smoke and betel nut chewing promoted the development of chronic inflammation reactions of oral tissue, which subsequently lead to accumulation of random genetic alteration and initiate the development of oral cancer [36, 37]. The polymorphonuclear granulocytes (PMNs) contributed to inflammatory activity of tumor microenvironment in head and neck cancer patients by secretion of CCL4, lactoferrin, and matrix metalloproteinase 9 [15]. We suggested tobacco smoking and betel nut chewing induce increased expression of CCL4 and result in augmented inflammatory response toward tumor promotion and deteriorate anti-tumor immunity.

As mentioned, smoking and betel nut chewing combination with CCL4 polymorphism can increased risk to rapid progress oral cancer; thus, a more active health education is needed in those patients with a higher CCL4L polymorphism. But we need further study to identify the association of CCL4 polymorphism and disease prognosis. The high serum chemokine level maybe help to detect oral cancer prognosis. A better understanding of genetic defects associated with oral cancer should provide to the development of novel therapies.

The linkage disequilibrium (LD) exists across the human genome and it can be used as genetic markers to locate adjacent variants that contribute to disease. The haplotype analyses can provide the contribution to the susceptibility of disease [38]. We evaluated impacts of different haplotype combinations of 2 CCL4 SNPs (rs1634507, rs10491121) to the risk of oral cancer and eventually found that the GG haplotype showed a high risk for OSCC (Table 5). It is possible that the GG haplotype of CCL4 is in LD with other functional polymorphisms that are responsible for the susceptibility to OSCC.

However, we also found that A/G genotype CCL4 rs10491121 polymorphism and GG haplotype combinations of CCL4 rs1634507/rs10491121 polymorphisms represented a protective factor for oral cancer development and progression. Patients with A/G genotype CCL4 rs10491121 correlated with a significantly lower tumor size and trend well cell differentiated grade compared with patients with A/A alleles. Alicia *et al*. showed that CCL4 secretion by CD4+- antigen-presenting cell (APC) complexes, which correlated with increased cross-priming of CCR5-expressing CD8+ T lymphocytes [39]. Then CD8+ T lymphocytes exit the draining lymph node and migrate to the tumor site, where they exert their cytotoxic function on cancer cells. High levels of CCL4 in the ESCC patients predicted prolonged survival. CDR5+CD8+ T lymphocytes were recruited by CCL4 into ESCC lesions. We suggested A/G genotype CCL4 rs10491121 polymorphism recruit different T lymphocyte subsets increasing anti-tumor immunity, which benefits the inhibition of tumor growth.

In conclusion, our results suggest that T allele of CCL4 rs1634507 G/T polymorphism may be associated with the susceptibility to oral cancer. The interactions of gene to oral cancer-related environmental carcinogen have a synergetic effect that can further promote oral cancer development. The GG haplotype of the 2 CCL4 SNPs (rs1634507, rs10491121) combined also enhance risk of OSCC. The CCL4 rs10491121 gene polymorphism may be a protective factor against oral cancer progression.

## MATERIALS AND METHODS

### Subjects collection

In 2007–2015, we recruited 861 male patients (mean age of 54.92 ± 11.04 years) with oral squamous cell carcinoma at Chung Shan Medical University Hospital in Taichung and Changhua Christian Hospital in Changhua, Taiwan as the case group. For the control group, 1192 health participants (mean age of 53.90 ± 10.01) without past history of cancer of any sites were included. These participants have visited those same hospitals and were from the same geographic area. Subjects with the oral precancerous disease such as oral submucous fibrosis, leukoplakia, erythroplakia, verrucous hyperplasia, etc. were excluded the control group. Including TNM clinical staging, the primary tumor size, lymph node involvement, and histologic grade, was obtained from their medical records. Oral-cancer patients were clinically staged according to the TNM staging system of the American Joint Committee on Cancer (AJCC). Tumor differentiation was examined by a pathologist according to the AJCC classification.

This study was approved by the Institutional Review Board of Chung Shan Medical University Hospital and informed written consent to participate in the study was obtained from each individual.

### Selection of CCL4 polymorphisms

In this study, the CCL4 SNPs were chosen belongs to the multiallelic CNVs [40] and extends across the q12 region of chromosome 17 that includes CCL4 genes using NCBI database. We selected the nonsynonymous SNPs (rs1634507, rs10491121, and rs1719153) for this study by NCBI SNP database. SNP with the CCL4 genes (rs1719153) was included in this study since this SNP was found to affect the CCL4 and HIV infection [21].

### Genomic DNA extraction

Genomic DNA was extracted from peripheral blood leukocytes using QIAamp DNA blood mini kits (Qiagen, Valencia, CA, USA) following the manufacturer's instructions. We dissolved DNA in a TE buffer (10 mMTris, 1 mM EDTA; pH 7.8) and it was subsequently quantified by measuring the OD260. The final preparation was stored at 220uC and then used to create templates for the polymerase chain reaction (PCR).

### Real-time PCR

Assessment of allelic discrimination for the CCL4 SNPs was done by the TaqMan assay with an ABI StepOne™ Real-Time PCR System (Applied Biosystems, Foster City, CA, USA), and further assessed with SDS version 3.0 software (Applied Biosystems). The primer and probes for analysis of the CCL4 gene polymorphisms were rs1634507 (product ID: C_7451708_10), rs10491121 (product ID: C_11626804_10) and rs1719153 (product ID: C_12120537_10). The total volume of TaqMan assays was 10 μL, containing 5 μL of Master Mix, 0.25 μL of probes, and 10 ng of genomic DNA. The real-time PCR reaction included an initial denaturation step at 95°C for 10 min, followed by 40 amplification cycles of 95°C for 15 secs and 60°C for 1 min.

### Statistical analyses

Differences between the 2 groups were considered significant if *p*-values were less than .05. Hardy-Weinberg equilibrium (HWE) was assessed using a chi-square goodness-of-fit test for biallelic markers. The Mann-Whitney U-test and Fisher's exact test were used to compare differences in the demographic characteristics between the healthy control and patients with oral cancer groups. The statistical analysis about haplotype was according to previously study [41]. The adjusted odds ratios (AORs) and 95% confidence intervals (CIs) for the association between genotype frequencies and the risk of oral cancer in addition to clinicopathological characteristics were estimated by multiple logistic regression models, after controlling for other covariates. All data was used the Statistical Analytic System software (v. 9.1, 2005; SAS Institute, Cary, NC, USA).
